# Association between sedentary and physical activity patterns and risk factors of metabolic syndrome in Saudi men: A cross-sectional study

**DOI:** 10.1186/s12889-015-2578-4

**Published:** 2015-12-12

**Authors:** Shaea Alkahtani, Ahmed Elkilany, Mohammed Alhariri

**Affiliations:** Department of Exercise Physiology, College of Sport Sciences and Physical Activity, King Saud University, PO Box 1949, Riyadh, 11441 Saudi Arabia; Department of Physical & Health Education, College of Preparatory Year & Supporting Studies, University of Dammam, Dammam, Saudi Arabia; Department of Physiology, College of Medicine, University of Dammam, Dammam, Saudi Arabia

**Keywords:** Actigraphy, Accelerometer, High-density lipoprotein, Metabolic syndrome

## Abstract

**Background:**

This study examined the association between objectively measured physical activity patterns and risk factors of metabolic syndrome (MetS) in Saudi men.

**Methods:**

The study was cross-sectional, and 84 healthy men from the Saudi population (age 37.6 ± 8.8 years, body mass index [BMI] 28.4 ± 5.4 kg/m^2^) were recruited. Measurements of physical activity were made using triaxial accelerometers over 7 consecutive days of leisure time physical activity. Waist circumference and blood pressure were measured, and fasting blood samples taken to measure glucose, high density lipoprotein cholesterol (HDL), and triglycerides (TG).

**Results:**

A total 21.4 % of participants had three or more risk factors for MetS, with low HDL levels the most frequent factor. Light physical activity (LPA) and BMI explained 13 % of the variation in TG. Moderate to vigorous physical activity (MVPA) with a minimum 10-min per session (10-min MVPA), LPA, and BMI explained 16 % of the variation in HDL. Sedentary behavior was not significantly associated with risk factors of MetS, although odds ratios indicated that decreased sedentarism does have a protective effect against MetS.

**Conclusions:**

LPA and 10-min MVPA were associated with elevated HDL levels among Saudi men. Future studies should confirm whether time spent physically active independent of intensity is an important factor in improving HDL levels.

## Background

Sedentary behavior is any activity during which energy expenditure is ≤ 1.5 metabolic equivalents (METs), such as sitting or standing. Sedentary behavior is a recognized independent risk factor of diabetes, metabolic syndrome (MetS), cardiovascular diseases (CVD), and mortality, independently of physical activity (PA) levels [1]. For example, mechanisms of the effect of sedentary behavior on lipoprotein and glucose levels include its direct effect on lipoprotein lipase and glucose transporter protein content, independent of PA [2]. A decrease in lipoprotein lipase has been suggested to underlie the correlation between increased LDL and sedentary behavior, independently of PA [3]. In addition, sedentary time was associated with 2-h glucose tolerance, triglyceride (TG), and high density lipoprotein cholesterol (HDL) levels in adults [2]. There is evidence for the association between prolonged sedentarism and adverse health outcomes, and prolonged sitting in particular is an emerging health risk. The likelihood of having high blood pressure, diabetes, heart disease, cancer, and combined chronic diseases are associated with sitting time, independent of PA and other potentially confounding factors [4]. A British longitudinal study found that sedentary behavior is associated with increased risk for all-cause mortality, which was lower in participants with non-sitting occupations and high levels of leisure time activity [5].

Guidelines for PA suggest that the minimum threshold for adults is to accumulate 10-min sessions of moderate-intensity PA, to reach 30 min, for at least 5 days a week and preferably every day [6]. Exercise intensity is an important factor for reversing the risk factors of MetS and improving aerobic capacity via different physiological mechanisms, including insulin action, endothelial function, and lipogenesis [7]. Both moderate and vigorous exercise intensities are associated with reduced risk of being classified as having MetS among middle-aged populations [8]. In locations with hot and humid weather, such as Saudi Arabia, some people may prefer to engage in low intensity levels of PA because of negative effects of the climatic conditions on exercise performance and physical capacity, particularly when intensity increases [9].

Light physical activity (LPA) has been characterized as activities with energy expenditure of 1.6–2.9 METs. There is evidence that LPA is inversely associated with risk factors of MetS, independent of moderate to vigorous physical activity (MVPA) [10]. Several studies have found associations between LPA and body composition, insulin resistance, and TG levels [11, 12]. LPA was shown to be associated with an improvement in insulin sensitivity in type 2 diabetes mellitus, which is involved in regulation of mitochondrial biogenesis and correlated with changes in skeletal muscle genes [13]. Increasing daily leisure time LPA is important for improving overall health by proportionally decreasing sedentary time [10]. The total volume of daily PA may be a better reference metric for health benefits than the number of minutes spent engaged in MVPA; this has been emphasized in recent years with increased use of objective measures of PA [14].

In the Gulf Cooperation Council (GCC), the prevalence of adults who are physically active for at least 150 min per week ranges between 39.0 and 42.1 % for men and between 26.3 and 28.4 % for women [15]. The prevalence of MetS in these countries ranges between 29.6 and 36.2 % for men and between 36.1 and 45.9 % for women. These rates are 10–15 % higher than in most developed countries, based on a systematic review using relevant studies [16]. A slightly higher prevalence of MetS has been found in Saudi Arabia, at 47.2 % among 2085 males aged between 20 and > 65 years; 85.6 % of participants had low HDL and 33.5 % had high waist circumference (WC) [17]. Other studies conducted using small populations have found lower prevalence of MetS among Saudi adults than the aforementioned studies. For example, one study found a prevalence of MetS among Saudi adults of 24.6 %; low HDL was the most frequent risk factor, present in 93 % of participants, whereas elevated fasting glucose was found in only 15.8 % of the study population [18]. Similarly, a MetS prevalence of 21 % was reported among 600 Saudi adults aged between 35 and 50 years, and this study did not find any association between PA level and the presence of MetS [19]. It is apparent that little research has been done in this field, and available data on MetS is sparse. High variation in MetS occurs between age groups, with interactions of sex and income [17]. Further studies investigating the prevalence of MetS and its risk factors among Saudi adults are warranted, together with accurate measures of PA patterns and their association with risk factors of MetS.

Most studies in the GCC region that investigated PA have recruited children or adolescents, used self-reported methods, and did not investigate the role of increased PA in reducing risk factors of MetS [19–21]. There is also a lack of studies investigating PA patterns in middle-income male office workers. A large body of research supports the use of motion sensors to measure changes in PA levels during lifestyle interventions [22–25]. There are three main types of motion sensors that can monitor daily leisure time activity: the pedometer, single-plane or uniaxial accelerometers, and triaxial accelerometers with measurement in three planes. The new model triaxial accelerometer from ActiGraph has several new functions that can help to determine PA patterns, including length and number of repetitions of sedentary behavior, LPA, and MVPA. The aim of the current study was to examine the association between objectively measured PA patterns and risk factors of MetS among Saudi male office workers with mid-level income.

## Methods

### Participant characteristics

Participants were men who met the following criteria: ≥ 20 years of age; non-athletes, defined as participation in regular exercise for competitive purposes; never diagnosed or treated for risk of MetS, according to the Adult Treatment Panel (ATP III) criteria[26]; non-smoker or having stopped smoking for more than 2 years; and having normal thyroid function and normal mobility. Participants were recruited from among staff and postgraduate students at the University of Dammam, teachers at four schools in Dammam and Al-Khobar in Saudi Arabia, and members of a community center in Dammam. The study targeted middle-income individuals who were office workers.

A total of 149 men agreed to participate in the current study. Written informed consent was provided by all participants. Initial measurements were taken, and accelerometers were given to participants. A total of 12 participants did not wear the accelerometer, and 39 participants did not meet the required device wearing time. Of the remaining 98 participants, 12 did not have any blood samples taken, and two participants reported in a follow-up questionnaire having been diagnosed with or treated for diabetes or high triglyceride levels. The total of included participants was 84 men (age, 37.6 ± 8.8 years; height, 172.8 ± 7.1 cm; weight, 85.0 ± 16.3 kg; and BMI, 28.4 ± 5.4 kg/m^2^); 16 of these participants were non-Saudis. The study protocol was approved by the Internal Review Board at the University of Dammam (IRB No. 2014-14-221).

### Study procedure

All participants were seated in air-conditioned rooms and had 3 to 5 min of rest before measurements were taken. After signing the consent form, height was measured to the nearest 0.5 cm and weight measured to the nearest 0.1 kg, using a digital stand scale fitted with a height column (model 27288; GIMA S.p.A., Gessate, Italy). WC was measured to the nearest 0.1 cm at the umbilicus using a tape measure. Participants were instructed to exhale while standing, and three WC measurements were taken after exhalation.

The average of three consecutive blood pressure readings with intervals of 5-min rest was obtained, using an electronic sphygmomanometer (Omron M6 Comfort HEM-7223-E; Omron Healthcare Co., Ltd., Kyoto, Japan). Participants were in a seated position with the arm supported at heart level, and systolic and diastolic blood pressure readings were recorded. At the end of this session, accelerometers were given to participants and referrals provided to a lab, for participants to have blood samples drawn.

### Data management of accelerometers

PA was measured using accelerometers (wGT3X-BT; ActiGraph LLC, Pensacola, FL, USA) for 7 consecutive days. Accelerometers were initialized for each participant, and all participants were asked to wear the device on their right hip throughout the day, except while sleeping and during water contact. Dependent variables obtained from the accelerometers were computed using ActiLife software (version 6.11.6; ActiGraph LLC). Validation of accelerometer wear time was computed using the algorithm of Troiano et al. [27] A period of nonwearing was when 60 min or more of vector magnitudes were determined to be zero (continuous inactivity), allowing 2 min of consecutive intervals with non-zero values less than 100 counts per minute. Vector magnitude thresholds were divided into five categories according to the Freedson cut point for adults as follows: sedentary; 0–99, light activity; 100–1951; moderate activity, 1952–5724; vigorous activity, 5725–9498; and very vigorous activity, 9499 and above. A minimum of 10 h of wear time per day for a minimum of 3 days (including 1 weekend day) was required to be considered as meeting the minimum inclusion criteria. The average wear time in the current study was 14.2 ± 1.5 h/day, for 5.3 ± 1.4 days.

### Data management of blood samples

Venous blood samples were collected from an antecubital vein at a private laboratory after overnight fasting of at least 10 h. Analyses of the blood samples were performed by a laboratory to assess HDL, TG, and plasma glucose levels.

Metabolic syndrome was defined according to ATP III criteria, based on having three or more of the following five risk factors: high WC (≥102 cm), elevated TG (≥150 mg/dL), low HDL (<40 mg/dL), high blood pressure (systolic ≥130 mmHg or diastolic ≥85 mmHg), and high blood glucose (≥110 mg/dL). MetS was also calculated using the criteria of a joint statement of several health organizations including the International Diabetes Federation (IDF). These criteria differ from those of the ATP III by using population and country-specific definitions of elevated WC (≥94 cm) and a glucose cutoff of ≥100 mg/dL [28].

### Statistical analysis

Data were analyzed using IBM SPSS for Windows, Version 20 (IBM Corp., Armonk, NY, USA). Data were presented as mean values and standard deviations. Descriptive statistics and frequency of daily PA and risk factors of MetS were calculated for both percentage and absolute values. An independent *t*-test was used to examine differences in risk factors of MetS based on sedentary time, LPA and MVPA. Binary logistic regression analysis was used to identify a significant impact of PA patterns on MetS status. Multiple linear regression was used to compute the model of significant independent variables of PA patterns on risk factors of MetS. A general linear model (univariate ANOVA) was used to assess the interaction between LPA and MVPA. An α-level of 0.05 was used to determine statistical significance.

## Results

Descriptive data of risk factors for MetS and PA patterns are shown in Table 1. A total 21.4 % of participants had three risk factors for MetS according to ATP III criteria, and the most frequent risk factor was low HDL. Analysis of MVPA for a minimum session of 10 min (10-min MVPA) showed that the average length of MVPA sessions was 11.4 ± 14.5 min; this was 20.8 ± 13.7 min when excluding participants who engaged in MVPA for less than 10 min.

**Table 1:** Descriptive data of risk factors for metabolic syndrome and physical activity patterns among Saudi men (*N* = 84)

Variables	No. (%)	Variables	No. (%)
BMI_2 (kg/m^2^)			
<25	19 (22.6)	≥25	65 (77.4)
BMI_4 (kg/m^2^)			
<20	4 (4.8)	25–29.9	42 (50)
20–24.9	13 (15.5)	≥30	23 (27.4)
Waist Circumference_ 94 (cm)		Waist Circumference_102 (cm)	
≥94	53 (63.1)	≥102	32 (38.1)
Glucose_100 (mg/dL)		Glocuse_110 (mg/dL)	
≥100	25 (29.8)	≥110	8 (9.5)
Blood pressure (mm Hg)		Triglycerides (mg/dL)	
≥130/85	13 (15.5)	≥150	23 (27.4)
HDL (mg/dL)			
<40	42 (50.0)	≥60	4 (4.8)
MetS (IDF)		MetS (ATP III)	
≥3 risk factors	28 (33.3)	≥3 risk factors	18 (21.4)
Daily Activity (Accelerometer)		MVPA (min/day)	
Sedentary (hr/day)	8.9 (61.9)	<30	45 (53.6)
LPA (hr/day)	4.9 (34.0)	≥30 - <60	29 (34.5)
MVPA(min/day)	34.2 (3.9)	≥60	10 (11.9)
10-min MVPA		20-min sedentary	
Bouts per week	1.7	Bouts per week	46.7
Average length (min/bout)	11.4	Average length (min/bout)	33.9
Participants who spent 0 bout	38 (46.4)	Time spent (hr/day)	4.9 (55.4)

No.: number of participants; %: percentage of total participants; BMI: body mass index; LPA: light physical activity; MVPA: moderate to vigorous physical activity; 10-min MVPA: moderate–vigorous physical activity with minimum of 10 min per session; 20-min sedentary: prolonged sedentary of ≥ 20 min per sedentary session; MetS: metabolic syndrome; IDF: International Diabetes Federation; ATP III: Adult Treatment Panel III at National Cholesterol Education Program

Data of MetS and PA patterns were divided into two subgroups (high and low), based on the mean of PA components (sedentary behavior, LPA, and MVPA). Table 2 shows the mean values and significant differences between these subgroups. Time spent engaged in sedentary behavior (h/day), time spent in prolonged sedentary behavior (h/day), and average length of prolonged sedentary behavior (min/session) were associated with age and were inversely associated with LPA.

**Table 2:** Differences in selected study variables based on daily activity patterns among Saudi men

	Sedentary (hr/day)		LPA (hr/day)		MVPA (min/day)		10-min MVPA (bout)	
	≥8.8	<8.8	≥4.8	<4.8	≥30	<30	≥1	<1
	(*n* = 41)	(*n* = 43)	(*n* = 41)	(*n* = 43)	(*n* = 39)	(*n* = 45)	(*n* = 46)	(*n* = 38)
Age	39.9	35.4	37.3	37.9	38.1	37.3	38.0	37.1
(years)	±10.2*	±6.6	±7.4	±10.0	±9.2	±8.6	±9.2	±8.5
BMI (kg/m^2^)	27.6	29.3	27.4	29.4	28.3	28.6	28.2	28.7
	±5.3	±5.4	±4.0	±6.2 ***	±4.7	±6.0	±5.5	±5.2
WC (cm)	95.9	99.4	94.2	100.7	97.7	97.8	97.1	98.3
	±14.3	±12.0	±11.4	±14.1*	±11.7	±14.6	±13.3	±13.3
Sedentary (hr/day)	NA	NA	8.4	9.2	8.8	8.9	8.8	8.9
			±1.3	±1.4**	±1.5	±1.3	±1.4	±1.4
LPA (hr/day)	4.5	5.1	NA	NA	4.9	4.7	4.8	4.8
	±0.8	±0.9**			±1.0	±0.8	±1.0	±0.9
MVPA (min/day)	33.5	34.8	35.5	32.9	NA	NA	42.8	23.7
	±19.4	±18.1	±16.1	±20.9			±19.0 **	±11.6
HDL (mg/dL)	41.1	42.5	44.2	39.5	41.7	42.0	43.6	39.7
	±8.5	±11.4	±11.1*	±8.5	±10.0	±10.4	±10.6***	±9.0
TG (mg/dL)	121.7	115.4	105.3	131.0	112.1	123.5	108.6	130.4
	±66.3	±70.1	±53.3	±78.0 ***	±64.7	±71.5	±68.5	±66.1
RHR (beat/min)	72.0	72.3	72.1	72.2	71.3	72.9	69.3	75.7
	±8.8	±8.5	±8.1	±9.1	±8.3	±8.9	±7.3	±8.9 *

*LPA*: light physical activity; *MVPA*: moderate to vigorous physical activity; 10-min *MVPA*: moderate–vigorous physical activity with minimum of 10 min per session; BMI: body mass index; *WC*: waist circumference; *HDL*: high density lipoprotein; *TG*: triglycerides; *RHR*: resting heart rate

* *p* ≤0.05; ** *p* ≤0.01; *** *p* = 0.08

After controlling for age and BMI, no PA patterns were significantly associated with MetS *(p  >*  0.05). However, odd ratios (OR) of 0.53 (0.15–1.82), 0.54 (0.17–1.75), and 0.58 (0.17–1.89) for MVPA, 10-min MVPA, and LPA, respectively, indicate that PA does have a protective effect against MetS. Likewise, although sedentary behavior is not significantly associated with the incidence of MetS, those who are likely to sit for more than 8.8 h/day are 2.02 (0.58–7.02) times more likely to develop MetS as compared with those who sit less than 8.8 h/day.

Multiple linear regressions for significant independent variables (LPA and 10-min MVPA) and dependent variables (HR, HDL, and TG) are shown in Tables 3 and 4. Univariate ANOVA revealed that there was no interaction between LPA or 10-min MVPA and HDL (*p* = 0.5).

**Table 3:** Stepwise linear regression model of significant independent variables on TG

Model	Independent variables	B	t	P	F	R^2^
1	BMI	48.0	−2.1	0.009	7.1	0.08
2	BMI	52.0	−2.2	0.004	6.3	0.13
	LPA	32.7	−2.2	0.02		

*TG* triglycerides; *BMI* body mass index; *LPA* light physical activity

**Table 4:** Stepwise linear regression model of significant independent variables on HDL

Model	Independent variables	B	t	P	F	R^2^
1	10-min MVPA	−4.7	−2.1	0.03	4.6	0.05
2	10-min MVPA	−4.7	−2.2	0.03	4.9	0.11
	LPA	−4.7	−2.2	0.03		

*HDL* high density lipoprotein; *LPA*: light physical activity, 10-min *MVPA*: moderate-vigorous physical activity with minimum length of 10 min per session

Multiple regression for age, BMI, and activity variables in predicting resting heart rate (RHR) showed that 10-min MVPA was the significant predictor of RHR, explaining 14 % of changes in RHR (*F* = 10.9, *p* = 0.001), and *B*-values were significant (*B* = 6.3, *p* = 0.001). In addition, LPA and 10-min MVPA were not significantly associated with changes in WC. Only BMI (*B* = 2.1, *p* = 0.001) and age (*B* = 0.2, *p* = 0.003) explained 79 % of changes in WC (*F* = 151.2, *p* = 0.001).

## Discussion

The aim of the current study was to examine the association between objectively measured PA patterns and risk factors of MetS in 84 middle-income male office workers from the Saudi population. The prevalence of MetS was 21.4 % as according to ATP III criteria. Low HDL was the most frequent risk factor, present in 50 % of participants. Daily leisure time activity among our study population comprised 66.2 % sedentary behavior, 28.5 % LPA, and 5.2 % MVPA. 10-min MVPA was the strongest factor that explained 5 % of the variation in HDL, and including LPA in the model explained 11 % of the variation in HDL. MVPA and sedentary patterns, including prolonged sedentary behavior with a minimum of 20 min per session, were not associated with variations in the risk factors of MetS, although the ORs indicated that increased PA and decreased sedentary behavior does have a protective effect against MetS.

Half of our study participants had low HDL, 50 % were overweight, 63 % had WC ≥ 94 cm, and 38 % had WC ≥ 102 cm. The least frequent marker was blood glucose ≥ 110 mg/dL at 9.5 %, and 15.5 % of the participants were hypertensive. The current data were similar to Saudi data showing increased prevalence of low HDL and higher WC [17]. The prevalence of MetS in the current study, with the most frequent risk factor being low HDL and the least frequent blood glucose, was identical to other studies conducted among Saudi adults, in which the prevalence of MetS among Saudi adults was 21 % [18, 19]. It is important that different populations had different levels of risk factors for MetS, such that the association between PA patterns and MetS risk may also differ. For example, the NHANES study in the United States showed that the most frequent risk factor for MetS was WC, found in 65.5 % of participants [29]. Among 483 healthy Japanese adults (age 47.9 years and BMI 25.6 kg/m^2^), 52.3 % were abdominally obese, 50.1 % were hypertensive, and 23.2 % had MetS [10]. Among 1451 healthy, nondiabetic Chinese men, 17.9 % had MetS and 43.8 % were hypertensive [30]. It is notable that hypertension was the most frequent contributor to MetS among East Asian populations, whereas WC was the most frequent contributor among Americans. From the current study and previous studies of the Saudi population, it is important to examine whether low HDL is the most frequent contributor of MetS among Saudis.

For the first time, PA patterns among Saudi adults are reported using objective measures. The data showed that the current cohort spent 66.2 % of daily leisure time engaged in sedentary behavior, 28.5 % in LPA, and 5.2 % in MVPA, with an average accelerometer wear time of 14 h/day. This was within the normal range of daily leisure time activity in comparison with previous studies conducted among adults. For example, NHANES found that adults spent 65 % (9.5 h) of total accelerometer wear time engaged in sedentary behavior [29]. Among 33 men (40.7 years), accelerometry measures were as follows: 573 min or 9.5 h (63.9 %) engaged in sedentary behavior, 282 min or 4.7 h (31.2 %) in LPA, and 53 min (5.9 %) in MVPA [31]. Two combined studies showed that sedentary time accounted for 71 % (10.3 h) of average accelerometer wear time (14.5 h), independent of average age in the two studies (32.9 and 63.7 years); however, the younger cohort spent a longer time engaged in MVPA (0.7 vs. 0.5 h/day) [2].

Our findings showed that sedentary behavior, including 20-min sessions, was not associated with variations in risk factors of MetS; this outcome was not in agreement with other studies. For example, total sedentary time and prolonged sedentary time of at least 20-min sessions were associated with higher insulin levels among Canadian adults [32]. Participants in the highest quartile of sedentary time were more likely to have MetS than those in the lowest quartile [33]. It is important that PA levels may not be correlated to sedentary behavior in a dose–response relationship. For example, sedentary time was inversely and moderately correlated with total PA and MVPA, but MVPA was strongly correlated with total PA [2]. It should be noted that variation in sedentary time among participants was very small. Recruiting individuals who engage in a wide range of PA levels from the Saudi community would better illustrate the association between sedentarism and risk factors of MetS. Further research is needed to determine the threshold length of sedentary behavior that is associated with cardiometabolic events, and to describe the types of sedentary behaviors involved.

The role of LPA in HDL and TG levels in the current study was in agreement with several previous studies. For example, NHANES showed that greater time spent engaged in LPA, independent from MVPA, was associated with lower odds of elevated TG, low HDL, and elevated WC and was not associated with higher glucose levels or blood pressure [34]. Independent of MVPA, the association between LPA with TG and lipid accumulation was significant after adjustment for all covariates [12]. The inverse association between LPA and sedentary in the current study was expected, and an inverse proportional association between LPA and sedentary time has been previously reported [10]. The association between LPA and positive outcomes of MetS, such as HDL and TG levels in the current study, should be considered in future studies, to examine the role of PA intensity in improving health among apparently healthy, sedentary men.

There were no significant differences in PA, body composition, and metabolic markers based on MVPA, whereas 10-min MVPA differentiated participants with respect to HDL level and RHR. These findings emphasize the importance of the current general guidelines for adults to accumulate 10-min sessions of MVPA versus the volume of MVPA [6]. Interestingly, several recent studies have suggested that volume of MVPA, whether sporadic or consecutive, is a key factor for improving metabolic markers. For example, a cross-sectional analysis of 2109 adults compared short and long sessions of MVPA, and showed that accumulated shorter MVPA sessions of less than 10 min each favorably influence cardiometabolic risk [35]. Other data from national surveys also found that regardless of whether MVPA was accumulated in 5- or 10-min sessions, cardiometabolic risk factors in children and adults were improved, which means that the volume of MVPA is more important than the duration per session [36, 37]. Approximately half of the current participants did not accumulate 10-min sessions of MVPA. This was also found among young men, with total 10-min MVPA sessions of 13.9 min and accumulated sporadic MVPA of 59.1 min [38]. The current study favors 10-min MVPA for improving risk factors of MetS, especially HDL levels.

Limitations of the current study were that women and very active Saudi adults were not investigated. In addition, variations in physical fitness may better explain the effect of PA patterns on risk factors of MetS. The current study did not discriminate sitting from standing sedentary time; accumulated research has found a link between long periods of sitting and cardiometabolic events. Energy balance was not examined, which is an important arm to understanding the obesity epidemic and its association with MetS. The current study investigated the association between physical activity patterns and risk factors of MetS among men who were office workers with mid-level income. Thus, caution is required regarding generalizability of the results. Future studies should use objective measures in a manner similar to the current study, preferably using a larger sample size.

## Conclusion

MetS was found in 21.4 % of the Saudi male office workers in our study, with the highest risk factor low HDL. 10-min MVPA and LPA were associated with high HDL, and LPA was associated with low TG. Future studies should confirm whether time spent physically active, independent of intensity, is important for improving HDL and TG levels.
